# Intra-operative gallbladder scoring predicts conversion of laparoscopic to open cholecystectomy: a WSES prospective collaborative study

**DOI:** 10.1186/s13017-019-0230-9

**Published:** 2019-03-14

**Authors:** Michael Sugrue, Federico Coccolini, Magda Bucholc, Alison Johnston, Contributors from WSES, Dimitrios Manatakis, Orestis Ioannidis, Stefano Bonilauri, Mahir Gachabayov, Arda Isik, Wagih Ghnnam, Vishal Shelat, Muyiwa Aremu, Rajashekar Mohan, Giulia Montori, Maciej Walędziak, Magdalena Pisarska, Victor Kong, Marcin Strzałka, Paola Fugazzola, Gabriela Elisa Nita, Matteo Nardi, Piotr Major, Ionut Negoi, Andrea Allegri, Georgios Konstantoudakis, Isidoro Di Carlo, Damien Massalou, Giuseppe D’Amico, Leonardo Solaini, Marco Ceresoli, Roberto Bini, Martin Zielinski, Matteo Tomasoni, Andrey Litvin, Belinda De Simone, Eftychios Lostoridis, Fernando Hernandez, Gabor Panyor, Gustavo M. Machain V, Ioanna Pentara, Luca Baiocchi, Kin Cheung Ng, Luca Ansaloni, Massimo Sartelli, Miguel Leon Arellano, Natalia Savala, Neville Couse, Sarah McBride

**Affiliations:** 1 Donegal Clinical Research Academy, Letterkenny University Hospital, Donegal, Ireland; 2 Papa Giovanni Hospital, Bergamo, Italy; 3 EU INTERREG Centre for Personalised Medicine, Intelligent Systems Research Centre, School of Computing, Engineering and Intelligent Systems, Ulster University, Magee Campus, Derry~Londonderry, Northern Ireland

**Keywords:** Cholecystitis, Operative severity scoring system, Conversion to open cholecystectomy, Index surgery, Surgical outcomes

## Abstract

**Introduction:**

Laparoscopic cholecystectomy, the gold-standard approach for cholecystectomy, has surprisingly variable outcomes and conversion rates. Only recently has operative grading been reported to define disease severity and few have been validated. This multicentre, multinational study assessed an operative scoring system to assess its ability to predict the need for conversion from laparoscopic to open cholecystectomy.

**Methods:**

A prospective, web-based, ethically approved study was established by WSES with a 10-point gallbladder operative scoring system; enrolling patients undergoing elective or emergency laparoscopic cholecystectomy between January 2016 and December 2017. Gallbladder surgery was considered easy if the G10 score < 2, moderate (2 ≦ 4), difficult (5 ≦ 7) and extreme (8 ≦ 10). Demographics about the patients, surgeons and operative procedures, use of cholangiography and conversion rates were recorded.

**Results:**

Five hundred four patients, mean age 53.5 (range 18–89), were enrolled by 55 surgeons in 16 countries. Surgery was performed by consultants in 70% and was elective in (56%) with a mean operative time of 78.7 min (range 15-400). The mean G10 score was 3.21, with 22% deemed to have difficult or extreme surgical gallbladders, and 71/504 patients were converted. The G10 score was 2.98 in those completed laparoscopically and 4.65 in the 71/504 (14%) converted. (*p* <  0.0001; AUC 0.772 (CI 0.719–0.825). The optimal cut-off point of 0.067 (score of 3) was identified in G10 vs conversion to open cholecystectomy. Conversion occurred in 33% of patients with G10 scores of ≥ 5. The four variables statistically predictive of conversion were GB appearance—completely buried GB, impacted stone, bile or pus outside GB and fistula.

**Conclusion:**

The G10 operative scores provide simple grading of operative cholecystectomy and are predictive of the need to convert to open cholecystectomy. Broader adaptation and validation may provide a benchmark to understand and improve care and afford more standardisation in global comparisons of care for cholecystectomy.

## Introduction

Laparoscopic cholecystectomy not only is the cornerstone of management of biliary disease and cholecystitis but is one of the commonest operations in both elective and emergency surgery. It offers an unquestionable advantage over open cholecystectomy to the patient and the health care system [1]. It is essential therefore that simple metrics can be applied to understanding the course of surgery and its outcome. While completion of the operation laparoscopically is not a proven quality indicator, analysis of surgical performance needs greater scrutiny [2–4]. Outcomes from cholecystectomy, particularly in terms of operative approaches and findings, use of intra-operative cholangiography, conversion from laparoscopic to open, length of surgery and morbidity, including readmission to hospital, vary. There are many variables in the management of cholecystitis, requiring a tailored approach due in part to the large heterogeneity of the patients and the actual state of the gallbladder at surgery. Interpreting the cause of and reducing this variability is a key to advancing outcomes following laparoscopic cholecystectomy.9 Conversion to open cholecystectomy is itself not only occasionally a necessity but a safer option than proceeding laparoscopically. Surgeons, with far greater exposure to laparoscopic technique, may opt for different damage control procedures rather than conversion to open, including various forms of bailout techniques [5].

It is important therefore that there is standardization of documentation and communication, with risk-adjusted measures, to allow qualitative studies and outcome comparisons. Accurate and reproducible stratification of the severity of gallbladder (GB) disease requires a scoring/grading system that is easily implemented, clinically and operatively relevant and simple. A number of publications have reported new scoring and grading systems [6–10]. Some of these scores are based on preoperative clinical findings, and imaging, but only concentrate on actual operative findings limiting their use. Recently, the AAST scoring system has been validated and it has been suggested that it is superior to the 2013 Tokyo classification in part due to the greater number of grades of cholecystitis with the AAST classification [11]. The Tokyo guidelines for classifying cholecystitis use three grades, without robust inclusion of the operative findings [12]. More recently, the Tokyo updates have expanded the potential scoring-grading system, but this remains yet to be validated.

As surgeons practising in both elective and emergency general surgery, we are well aware that the operative findings and difficulty hold the key to outcome.

We reported a 10-point operative scoring system of cholecystitis severity to facilitate a potential benchmark for international analysis [7].

This study undertook a prospective evaluation of a recently reported intra-operative G10 gallbladder scoring system to determine if it could predict the outcome of surgery, primarily the ability to complete the operation laparoscopically.

## Methods

A prospective ethically approved multicentre study was undertaken, between January 2016 and December 2017, under the leadership of the World Society of Emergency Surgery. An open invitation was sent to surgeons to register and enrol their patients undergoing laparoscopic cholecystectomies, either as elective or emergency procedures. Data was entered in a web-based data entry sheet [13]. Surgeons registered on-line and then enrolled their de-identified patient data, after completion of surgery, to include demographics, patient age, gender, nature of surgery either elective or emergency surgery, into a 10-point intra-operative gallbladder scoring system (G10) (Table 1). The G10 cholecystitis severity score focuses on four key components: the gallbladder’s operative appearance, whether distended or contracted, ease of access and the presence of sepsis in the peritoneal cavity, either biliary peritonitis or purulent fluid, and/or a cholecysto-enteric fistula. The scoring system differed very slightly from Sugrue’s original published 10-point operative score with the addition of an extra category for the degree of gallbladder adhesions (scoring 2 points). The previous (single point) score for time to identify the cystic artery and duct was removed and replaced with a category which considered limited access due to adhesions from previous surgery.

**Table 1:** Cholecystitis severity score used for G10

Cholecystitis severity	Score
Appearance	
Adhesions < 50% of GB	1
Adhesions> 50% but GB buried	2
Completely buried GB	3 (max)
Distension/contraction	
Distended GB or contracted shrilled GB	1
Inability to grasp without decompression	1
Stone > 1 cm impacted in Hartmann’s pouch	1
Access	
BMI > 30	1
Adhesions from previous surgery limiting surgery	1
Sepsis and complications	
Free bile or pus outside the gallbladder	1
Fistula	1
Total possible	10

Further information was recorded relating to the occurrence of intra-operative complications, use of intra-operative cholangiography (IOC), and previous intervention of the common bile duct (CBD). The surgeons documented whether the procedure was completed laparoscopically or converted to open. In addition, the definitive type of cholecystectomy performed either total or subtotal cholecystectomy was noted. The operative time and level of experience of the surgeon was recorded. Patient identifiers were limited to the patients’ age, date of procedure, the email of the surgeon and the Centre. The relationship of the surgical volume to open conversion was explored. A non-parametric Spearman test was used to assess the strength of the relationship between the number of operations per-consultant and percent converted from laparoscopic to open cholecystectomy.

Gallbladder surgery was considered easy if the G10 score < 2, moderate (2 ≦ 4), difficult (5 ≦ 7) and extreme (8 ≦ 10).

Descriptive data was presented as mean, standard deviation and range. Mann-Whitney *U* test was used to evaluate the significance of differences between continuous variables. Fisher exact test was used to find the significant association between the G10 score and the outcome. A *p* value < 0.05 represented statistical significance. Univariate analysis was performed to identify risk factors associated with conversion to open cholecystectomy. Variables with a *p* value < 0.1, i.e. GB appearance, adhesions from previous surgery, impacted stone, bile or pus outside GB, distended or shrivelled GB, inability to grasp without decompression and fistula, were considered clinically relevant (Table 2) and entered into the logistic regression model. The accuracy of G10 to predict conversion to open cholecystectomy was assessed using the area under the receiver operating curves (AUR) with 95% confidence intervals (CI). ROC curve and its area under the curve (AUC) were calculated for the accuracy in predicting the outcome (i.e. no conversion to open cholecystectomy vs conversion to open cholecystectomy) based on G10 scores.

**Table 2:** G10 score and conversion rates

G10 score	Conversion to open cholecystectomy (%)	
	No	Yes
1	96.6	3.4
2	97.5	2.5
3	87.6	12.4
4	81.7	18.3
5	70.4	29.6
6	66.7	33.3
7	68.4	31.6
8	33.3	66.7

## Results

Five hundred four patients, mean age 53.5 (range 18–89), were enrolled by 55 surgeons in 16 countries. Two hundred ninety-five out of five hundred four (58.5%) were female and 284/504 (56.3%) were over the age of 50 years. Surgery was elective in 281/504 (56%). The mean number of laparoscopic cholecystectomies each surgeon performed was 9.2 ± 12.9, (range 1–63). The mean conversion rate to open surgery was 14.3%, range 0–100%. The conversion rate was 7.5% in elective and 22.4% in emergency cases. The conversion rate was 15.1% for surgeons performing ≥ 5 cases. Surgery was performed by consultants in 353/504 (70%) of which 57% was elective, compared to 66% for residents. The mean operative time was 78.7 min (range 15–400). This was 71.8 min (15–400) for elective and 87.3 min (24–278) for emergency cases respectively (*p* ≤  0.0001).

Minor adhesions to the GB (covering < 50% of gallbladder) were found in 94/223 (42.4%) emergency and 192/281 (68.3%) in elective cases. GB adhesions > 50% occurred in 64/223 (28.7%) emergency compared to 67/281 (23.8%) elective and completely buried in 65/281 (23.1%) emergency compared to 22/281 (7.8%) in elective cases.

A distended or contracted/shrivelled gallbladder was found in 118/223 (52.9%) of emergency surgeries compared to 105/281 (37.4%) of elective (*p* = 0.004). Similarly, bile and pus indicative of evolving biliary peritonitis were found in 61/223 (27.4%) of emergency cases and 5/281 (1.8%) of elective (*p* = 0.0001). The relationship between risk factors and conversion are shown in Table 2. Univariate analysis of risk factors for conversion is shown in Table 3. Following multivariate analysis factors predictive of conversion to open cholecystectomy included a completely buried gallbladder, a stone impacted in Hartmann’s pouch, biliary peritonitis and a fistula (Table 4). Overall, 112/504 (22.2%) patients were found to have a difficult or extreme degree of operative difficulty as judged by a G10 score of 5 or greater.

**Table 3:** Risk factors for conversion to open cholecystectomy in univariate analysis

Risk factor	OR (95%CI)	*p* value
Gallbladder (GB) appearance	3.43 (2.38, 4.94)	< 0.0001***
BMI	1.04 (0.64, 1.68)	0.891
Adhesions from previous surgery limiting access	3.14 (1.71, 5.75)	0.0005***
Distended or shrivelled GB	1.72 (1.16, 2.55)	0.0018**
Inability to grasp GB	1.92 (1.25, 2.95)	0.0013**
Stone > 1 cm impacted in Hartmann’s pouch	2.14 (1.39, 3.3)	0.0002***
Bile or pus outside GB	3.99 (2.33, 6.83)	< 0.0001***
Fistula	10.5 (2.48, 44.43)	0.0019**

** - statistically significant at the 0.01 level

*** - statistically significant at the 0.001 level

**Table 4:** Risk factors for conversion to open cholecystectomy in multivariate

Risk factor	Level	Outcome		Multivariate odds ratios (95% CIs)	*p* value
		No conversion to open cholecystectomy no. (%)	Conversion to open cholecystectomy no. (%)		
Gallbladder (GB) appearance	Adhesions covering < 50% of GB	264 (92.3%)	22 (7.7%)		
	Adhesions > 50% but GB visible	111 (84.7%)	20 (15.3%)	1.41 (0.71, 2.82)	0.3264
	Completely buried GB	58 (66.7%)	29 (33.3%)	2.50 (1.17, 5.33)	0.018*
Adhesions from previous surgery limiting access	No	397 (88%)	54 (12%)	2.05 (0.99, 4.25)	0.055
	Yes	36 (67.9%)	17 (32.1%)		
Distended or shrivelled GB	No	254 (90.4%)	27 (9.6%)	1.52 (0.86, 2.69)	0.1508
	Yes	179 (80.3%)	44 (19.7%)		
Inability to grasp GB	No	311 (89.4%)	37 (10.6%)	1.30 (0.72, 2.33)	0.3796
	Yes	122 (78.2%)	34 (21.8%)		
Stone > 1 cm impacted in Hartmann’s pouch	No	322 (89.7%)	37 (10.3%)	1.96 (1.09, 3.55)	0.0257*
	Yes	111 (76.6%)	34 (23.4%)		
Bile or pus outside GB	No	391 (89.3%)	47 (10.7%)	2.75 (1.37, 5.53)	0.0046**
	Yes	42 (63.6%)	24 (36.4%)		
Fistula	No	430 (86.7%)	66 (13.3%)	9.14 (1.85, 45.16)	0.0066**
	Yes	3 (37.5%)	5 (62.5%)		

* - statistically significant at the 0.05 level

** - statistically significant at the 0.01 level

Operative cholangiograms were performed in 68/504 (13%). Prior ERCP was performed in 79/504 (16%).

The overall mean G10 score was 3.2 ± 1.7 and 3.0 ± 1.6 in the 433/504 (85.9%) completed laparoscopically and 4.7 ± 1.7 in the 71/504 (14.1%) converted (*p* = 5.274e−10, *p* <  0.0001; AUC (95% CI) was 0.772 (0.719–0.825). By maximizing sensitivity + specificity across various cut-off points, the optimal cut-off point of 0.067 (G10 = 3) was identified in G10 vs conversion to open cholecystectomy.

Conversion occurred in 33% of patients with G10 scores of ≥ 5. Thirty patients were reported as having intra-operative complications 22/30 (73%) occurring in the easy or moderate disease severity category.

A Fisher *p* value = 5.274e−10 shows that G10 score is significantly associated with the conversion to open cholecystectomy.

**Fig. 1:**
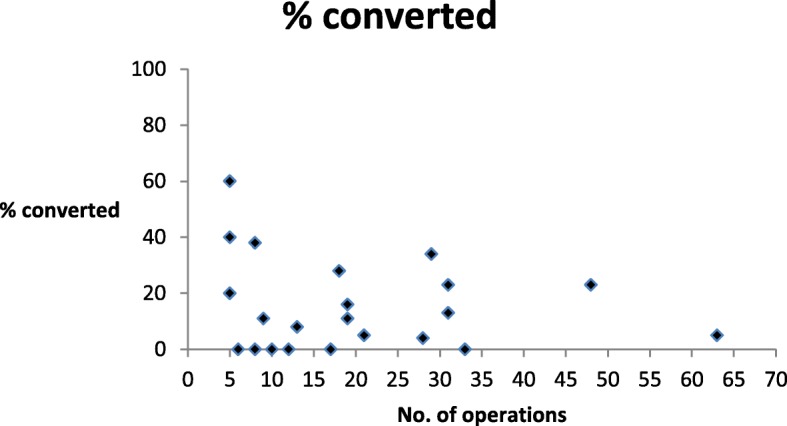
The relationship between the number of cholecystectomies performed and conversion, (rho = −0.17)

The relationship between the number of cholecystectomies performed and conversion is shown in Fig. 1 with conversion rates higher in those undertaking smaller numbers. The correlation coefficient rho = − 0.17 suggests a negative, but relatively weak, correlation between these two variables—implying a higher conversion rate for individuals performing fewer operations.

Table 5 shows an analysis of outcome with risk factors for intra-operative complications.

**Table 5:** Prevalence of risk factors in those with and without complications

Risk factors	Level	Outcome	
		No intraoperative complications no. (%)	Intraoperative complications no. (%)
Gallbladder (GB) appearance	Adhesions covering < 50% of GB	274 (95.8%)	12 (4.2%)
	Adhesions > 50% but GB visible	120 (91.6%)	11 (8.4%)
	Completely buried GB	80 (92%)	7 (8%)
BMI	≦ 30	325(94.2%)	20 (5.8%)
	> 30	149 (93.7%)	10 (6.3%)
Adhesions from previous surgery limiting access	No	424 (94%)	27 (6%)
	Yes	50 (94.3%)	3 (5.7%)
Distended or shrivelled GB	No	260 (92.5%)	21 (7.5%)
	Yes	214 (96%)	9 (4%)
Inability to grasp GB	No	330 (94.8%)	18 (5.2%)
	Yes	144 (92.3%)	12 (7.7%)
Stone 1 cm impacted in Hartmann’s pouch	No	342 (95.3%)	17 (4.7%)
	Yes	132 (91%)	13 (9%)
Bile or pus outside GB	No	415 (94.8%)	23 (5.2%)
	Yes	59 (89.4%)	7 (10.6%)
Fistula	No	467 (94.2%)	29 (5.8%)
	Yes	7 (87.5%)	1 (12.5%)

## Discussion

There is an unquestionable unmet need for robust, reproducible metrics to allow understanding of disease severity in patients with cholecystitis [14]. Defining the status of the gallbladder at surgery and the degree of any cholecystitis will facilitate more standardised reporting and improve pathways and management of risk-adjusted outcomes [15, 16]. Since Carl Langenbuch reported the first open cholecystectomy in 1882 and Muhe the first laparoscopic cholecystectomy in 1985 surprisingly it is only recently there has been increasing attention to grading severity of cholecystitis [7, 17]. There is now an agreement that we need to gain insight into the heterogeneity of cholecystitis and variance in outcome [18]. In the 1980s and 1990s, Hanna et al. and Nassar et al. described simple scales of difficulty for cholecystectomy [19, 20]. When we reported the G10 operative scoring system in 2015, we identified 16 published GB grading systems. Since then, there has been a number reported [10]. Confounding the variability of operative findings are paradigm shifts in the management of biliary disease [21, 22].

The cholecystectomy rate varies geographically, but is generally undertaken in between 100 to 200 per 100,000 inhabitants [23]. In the UK, 41% of patients have been admitted with prior cholecystitis before their eventual cholecystectomy [24].

Other large series have somewhat similar conversion rates although Hu and colleagues report only a 4% conversion rate [6, 11, 25]. There is extreme variability in the conversion rate in our study, and there is no reason to believe this does not reflect true practice. Even if we exclude surgeons who contributed less than five cases, the variability in conversion is from 0 to 60% with an average of 14%. As the volume of surgery increased, the conversion rate decreased. A weakness of this study was that we cannot be sure that all surgeons enrolled consecutive patients. This issue begs questions about surgical skills and techniques used to facilitate the safe completion of laparoscopic cholecystectomy. It highlights the potential needs for data registries and key performance indicators to provide meaningful analysis [4, 18, 26]. This would help both with surgical training and patient safety. This is crucially important with morbidity rates approaching 30%, and mortality of 5% after cholecystectomy, especially if the emergency cohorts are included.

With the ability to predict pre-operative difficult surgery, perhaps surgeons may increasingly opt out, explaining in part the increasing use of percutaneous cholecystostomy [27]. Almost 20% of patients in Hall’s cohort underwent PC cholecystostomy, with almost four times the complication rate of those undergoing emergency laparoscopic cholecystectomy. This needs to be balanced however by selection bias, potentially including more seriously ill patients in the cholecystostomy route [28]. Future research relating to conversion from laparoscopic to open cholecystectomy should report the percutaneous cholecystostomy rate. Previous suggestions that PC cholecystostomy is a desirable alternative to cholecystectomy are now in doubt [29]. The AUC for the G10 was 0.772 which is less than Hu’s recent reported Cairns Prediction Model with an AUC of 0.87 [25]. Their prediction model utilised three ultrasonographic and two clinical parameters. It is a pre-operative grading system. External validation had taken place with both Sutcliffe’s and Goonawardena’s predictive models with good AUC outcomes, only falling from 0.81 to 0.77 and 0.97 to 0.87 respectively [9, 30].

The current study has a number of limitations in part due to the desire to ensure simplifying surgeons involvement and ensure a broad ethically agreed international input. The question as to whether this is a validation study or development of a new score is important, but the changes between the first study in terms of scoring criteria were limited but need to be noted. Ideally developing a scoring system needs two stages, the development and its validation, and a much broader validation would be ideal. Surgeons when enrolling were not asked if their cases were consecutive nor asked to report exclusions. Furthermore, subjective opinion of the operating surgeon was accepted when grading the gallbladder appearance. Unlike other studies, photographic documentation of intra-operative findings was not required and operative pictures and videos were not uploaded or analysed [10]. This may have introduced bias in the study, but given the complexity of organising and ethical issues in storing patient data from 16 countries, this was not done. Inter-observer error when grading adhesions which limit surgery is rather subjective, and inter-observer variability has been reported in other laparoscopic assessment and grading, with a recent study in appendicitis showing poor reproducibility [31]. Our study only requested whether an intra-operative complication occurred or not and further descriptors were not requested. This prohibited significant analysis but was utilised to encourage engagement of surgeons as it was felt that underreporting would occur with surgeons reticent to enter this data into an international database. This reluctance has recently been reported with significant underreporting by surgeons of their intra-operative complications [32].

A key to optimising outcomes in cholecystectomy is a laparoscopic approach, albeit with a slightly increased risk of bile duct injury, and the latest Tokyo consensus emphasize that conversion to open is not a complication and in fact may be safer than pursuing the laparoscopic route in individual cases [5]. Bailout is an important option as surgeons may not possess the experience required for a complex open case. Conversion is not always a crime [33].

Grading systems have identified risk factors for both prolonged surgery and increased need for conversion. Wakabayashi et al. identified 19 operative risk factors potentially contributing to conversion [5]. As surgeons, we know that there are unique variable technical difficulties encountered during cholecystectomy and these fundamentally are related to the access, adhesion density and vascularity and the thickness, friability and weight and thickness of the gallbladder [34]. Recently, Wakabayashi et al., as part of the Tokyo 2018 guidelines, suggested 25 operative findings with scores that may affect the technical difficulty of cholecystectomy [5]. While we would disagree with Lee’s statement that there is no organised operative grading system, this study, and our previous study, suggests that the grading or scoring systems can be improved even further [7, 35].

The G10 score itself, while easy to perform, was not validated by independent photographic assessment or review of operative data. Unquestionably, there is a need for stratification of gallbladder severity in patients undergoing cholecystectomy. The Tokyo guidelines, like others, have not focused on operative finding when reporting outcomes [36]. The G10 score which was developed to anticipate conversion rate was therefore not used to study complications in this paper.

A disadvantage of the G10 is that it is an operative scoring system and patients who have interventions without surgery cannot be assessed. Patients undergoing percutaneous cholecystostomy who subsequently undergo surgery can be included. Given the mortality of high-risk cholecystitis patients, this needs to be addressed. This might help establish recent suggestions that percutaneous cholecystostomy is inferior to cholecystectomy [27]. The current is one of the largest reported prospective studies and adds to the debate about the benefits of both scores and grades in cholecystitis [34].

## Conclusion

This study has identified the need for greater understanding of conversion rates and readmission rates. The international surgical community needs to come to grips with the metrics of cholecystitis and cholecystectomy. The adoption of an agreed peri-operative grading or score of gallbladder disease and surgery is essential to advance the road to improved outcomes for our patients with biliary disease. The optimal cut-off point was a G10 score of 3 to predict conversion to open cholecystectomy. Conversion occurred in 33% of patients with G10 scores of ≥ 5. The four variables statistically predictive of conversion were GB appearance—completely buried GB, impacted stone, bile or pus outside GB and fistula. The G10 operative scores provide simple grading of operative cholecystectomy and are predictive of the need to convert to open cholecystectomy. Broader adaptation and validation may provide a benchmark to understand and improve care and afford more standardisation in global comparisons of care for cholecystectomy.

## Abbreviations

CBD: Common bile duct
ERCP: Endoscopic retrograde cholangiopancreatography
GB: Gallbladder
IOC: Intra-operative cholangiography
WSES: World Society of Emergency Surgery
